# Protection against High-Dose Highly Pathogenic Mucosal SIV Challenge at Very Low Serum Neutralizing Titers of the Antibody-Like Molecule CD4-IgG2

**DOI:** 10.1371/journal.pone.0042209

**Published:** 2012-07-27

**Authors:** Pascal Poignard, Brian Moldt, Karla Maloveste, Noëlie Campos, William C. Olson, Eva Rakasz, David I. Watkins, Dennis R. Burton

**Affiliations:** 1 Department of Immunology and Microbial Science and IAVI Neutralizing Antibody Center, The Scripps Research Institute, La Jolla, California, United States of America; 2 International AIDS Vaccine Initiative, New York, New York, United States of America; 3 The Centre d'Immunologie de Marseille-Luminy, and Institut National de la Santé et de la Recherche Medicale, and Centre National de la Recherche Scientifique, Marseille, France; 4 Progenics Pharmaceuticals, Inc. Tarrytown, New York, United States of America; 5 Department of Pathology and Laboratory Medicine, University of Wisconsin-Madison, Madison, Wisconsin, United States of America; 6 Department of Pathology, University of Miami, Miami, Florida, United States of America; 7 Ragon Institute of Massachusetts General Hospital, Massachusetts Institute of Technology, and Harvard, Boston, Massachusetts, United States of America; Harvard Medical School, United States of America

## Abstract

Passive transfer studies using monoclonal or polyclonal antibodies in the macaque model have been valuable for determining conditions for antibody protection against immunodeficiency virus challenge. Most studies have employed hybrid simian/human immunodeficiency virus (SHIV) challenge in conjunction with neutralizing human monoclonal antibodies. Passive protection against SIV, particularly the pathogenic prototype virus SIVmac239, has been little studied because of the paucity of neutralizing antibodies to this virus. Here, we show that the antibody-like molecule CD4-IgG2 potently neutralizes SIVmac239 in vitro. When administered by an osmotic pump to maintain concentrations given the short half-life of CD4-IgG2 in macaques, the molecule provided sterilizing immunity/protection against high-dose mucosal viral challenge to a high proportion of animals (5/7 at a 200 mg dose CD4-IgG2 and 3/6 at a 20 mg dose) at serum concentrations below 1.5 µg/ml. The neutralizing titers of such sera were predicted to be very low and indeed sera at a 1∶4 dilution produced no neutralization in a pseudovirus assay. Macaque anti-human CD4 titers did develop weakly at later time points in some animals but were not associated with the level of protection against viral challenge. The results show that, although SIVmac239 is considered a highly pathogenic virus for which vaccine-induced T cell responses in particular have provided limited benefit against high dose challenge, the antibody-like CD4-IgG2 molecule at surprisingly low serum concentration affords sterilizing immunity/protection to a majority of animals.

## Introduction

In the absence of an effective vaccine against HIV, it is pertinent to explore conditions under which immune mechanisms provide benefit against exposure to virus. For humoral immunity, this has mostly been done through observations of the ability of passively administered monoclonal and polyclonal neutralizing antibodies to protect against SHIV challenge in macaques [1], [2], [3], [4], [5], [6], [7], [8], [9]. Most studies have associated antibody protection with relatively high serum neutralizing titers [5], [10] although exceptions have been noted [2], [7], [8]. In particular, the broadly neutralizing human monoclonal antibody (bnMAb) 2G12 has been shown to provide protection against both X4 and R5 high-dose SHIV challenge at relatively low serum neutralizing antibody titers [2], [7]. Protection against repeated low-dose mucosal SHIV challenge has also been observed at notable lower serum neutralizing antibody titers than those typically required for protection against high-dose mucosal challenge [8].

SHIV models have continued to be employed in studies on humoral immunity while they have been criticized in studies on cellular immunity [11], [12], where they are much less used than several years ago. The focus of criticism with respect to “T cell vaccine” studies has been the failure of SHIV infections, particularly SHIV89.6P, to reproduce many features of HIV infection in contrast to SIV infection [11], [12]. T cell vaccines do not generally prevent infection but rather control infection once established. On the other hand, humoral immunity studies generally seek to provide sterilizing immunity, particularly against R5 SHIVs, and differences between SHIV and HIV infection are not given an opportunity to emerge. Consequently, antibody protection studies using SHIVs have been more readily accepted in recent years [13].

Nevertheless, researchers would like to have more data on the conditions for antibody protection against SIV. One recent study suggested that very low levels of neutralizing antibody induced through vaccination correlated with protection against low-dose repeated SIVsmE660 mucosal challenge [14]. Another study suggested that non-neutralizing or undetectable levels of neutralizing antibody were correlated with vaccine protection against low-dose repeated SIVmac251 challenge [15]. The most efficient protection against SIV infection has been achieved with live attenuated SIVmac239 delta Nef vaccination [16]. Interestingly, recent data has indicated that antibodies may play a role in the mucosal part of the protection against viral infection (Li, Haase et al, manuscript submitted). For safety concerns, the use of live attenuated virus may not be transferable into human vaccination strategies [17]. However, the level of protection induced serves as a “gold standard” and emphasizes the need for further investigation into conditions where protection against pathogenic SIV infection is achievable. As regards passive transfer studies, no potent monoclonal antibodies that neutralize SIVmac239, in particular, have been described, limiting the ability to conduct such studies.

In routine studies, we noted that SIVmac239 was sensitive to neutralization by the antibody-like molecule CD4-IgG2 in a classical PBMC assay. Although this molecule is not an antibody it is an entry inhibitor that neutralizes virus in classical assays and was available in larger amounts for animal protection studies. The molecule is a heterotetramer consisting of two chains of a CD4-IgG2 heavy chain fusion protein and two chains of a CD4-human kappa light chain fusion protein [18]. In each case, the membrane distal domains 1 and 2 of CD4 replace the variable domains of the IgG molecule to produce a molecule that is tetrameric with respect to CD4 binding activity. CD4-IgG2 is predicted to have 4 gp120 binding sites per molecule and thus potentially have higher avidity for HIV-1 virions or infected cells than monomeric soluble CD4. Indeed a number of studies demonstrated that CD4-IgG2 neutralizes a broad array of primary HIV-1 in in vitro and ex vivo neutralization assays with a potency comparable to that of human neutralizing mAbs such as b12, 2G12 and 2F5 [18], [19], [20]. As predicted the tetrameric molecule is far more potent than monomeric CD4. It appears that CD4-IgG2 may function, at least in part, like anti-CD4 binding site antibodies in inhibiting virus attachment [21], perhaps through inducing gp120 shedding [22].

The in vivo activity of the molecule was first demonstrated in the hu-PBL-SCID mouse model [23]. Protection was observed in a dose dependent manner that was directly correlated to neutralization as for human mAbs. Thus sterile protection against challenge with the T cell line adapted virus LAI was found for 9/9 mice at 10 mg/kg antibody corresponding to a serum antibody concentration at the time of challenge of about 250 times the 90% in vitro PBMC neutralization titer. Sterile protection against two primary HIV-1 was more difficult to achieve; 4/5 mice were protected at 50 mg/kg antibody corresponding to serum concentrations 30–60-fold greater than the IC_90_s for each isolate.

Phase 1/2 clinical studies showed that CD4-IgG2 is well tolerated at doses up to 25 mg/kg with a half-life of 2–4 days [24], [25], [26]. No patients developed antibodies to CD4-IgG2. In both infected children and adults with advanced disease, evidence was obtained of significant reductions in viral load for several weeks following treatment.

Here, we investigated the ability of CD4-IgG2 to protect against SIVmac239 mucosal challenge in macaques. Preliminary studies showed a relatively short half-life for the protein in macaques. Accordingly, in order to attempt to maintain CD4-IgG2 levels following viral challenge, we decided to deliver the protein by continuous infusion using an osmotic pump. We reasoned that this would more accurately reflect the conditions of relatively constant antibody concentration that would follow vaccination. The results show that the CD4-IgG2 molecule, even at relatively low serum concentrations, is associated with protection against SIVmac239 challenge in a majority of animals.

## Results

### Neutralization of SIVmac239 by CD4-IgG2

To investigate the ability of CD4-IgG2 to neutralize SIVmac239 in vitro, we tested the antibody-like molecule in three different neutralization assays (see Materials and Methods for description). First, in a macaque PBMC-based assay using replication competent SIVmac239, we found an IC_90_ of 1 µg/ml by fitting the data using nonlinear regression. Second, in an U87-cell-based assay using pseudovirus SIVmac239, the IC_50_ and IC_90_ were found to be 0.03 and 0.4 µg/ml. Third, in a CEM-cell-based assay using pseudovirus SIVmac239, an IC50 was found to be 0.06 µg/ml. No neutralization was observed in any of the neutralization assays using a non-SIV control IgG antibody. The assays demonstrate that SIVmac239 is sensitive to neutralization by CD4-IgG2.

### Protection of macaques against SIVmac239 challenge with CD4-IgG2

Preliminary studies of CD4-IgG2 in macaques showed that the pharmacokinetics of the molecule are roughly equivalent for subcutaneous, intravenous (i.v.) and intramuscular (i.m.) administration after day 1, although i.v administration does produce a high initial level of protein. Owing to the relatively rapid decay of CD4-IgG2, we chose to deliver it subcutaneously and continuously by an osmotic pump over a period of 14 days beginning 3 days before virus challenge.

**Figure 1:**
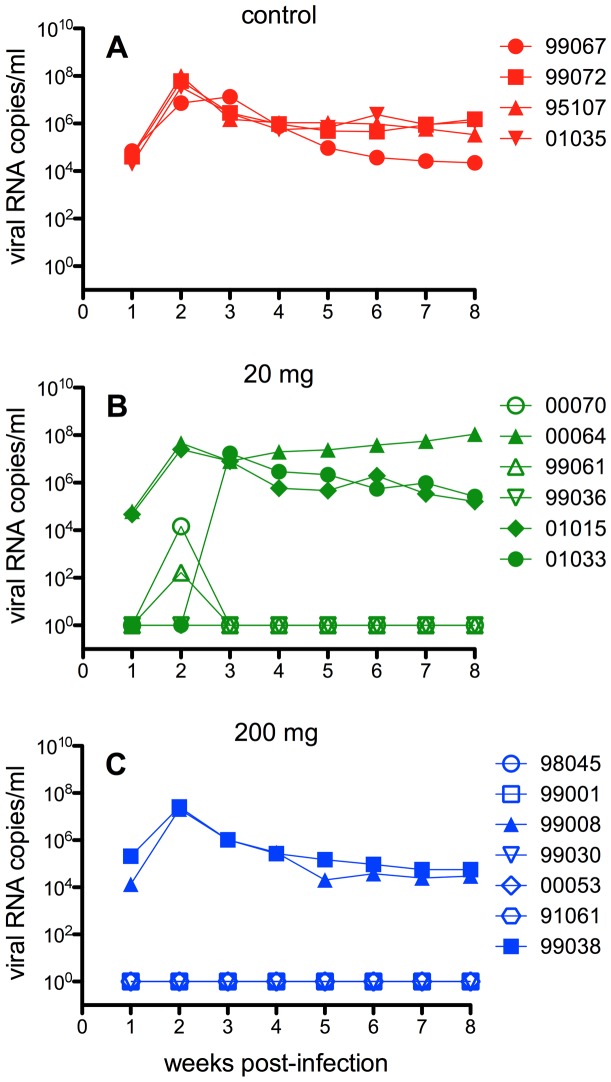
Protection of CD4-IgG2-treated rhesus macaques in a high-dose SIVmac239 challenge experiment. To maintain serum concentrations, CD4-IgG2 (or control human polyclonal IgG) was administered subcutaneously over a two-week period by an ALZET osmotic pump. Animals were challenged intrarectally with a single high dose inoculum (3–5×10^3^ TCID_50_) of SIVmac239 3-days after initiation of CD4-IgG2 administration. (**A**) Viral loads for animals treated with 200 mg of control polyclonal human IgG as a function of time following SIVmac239 challenge. All control animals became infected. (**B**) Viral loads for animals administered 20 mg CD4-IgG2 as a function of time following SIVmac239 challenge. Three out of 6 animals were fully protected and one infected animal showed delayed primary viremia. Due to a technical problem with the ALZET osmotic pump, one of the protected animals (98045) did not receive the full dose of 20 mg but this animal did not become infected. (**C**) Viral loads for animals administered 200 mg CD4-IgG2 as a function of time following SIVmac239 challenge. Five out of 7 animals were protected and showed no sign of infection at any time point. The minimum detection level was 125 SIV RNA copies/ml with a 95% confidence level. Open symbol indicates protected animal, closed symbol indicates infected animal.

The protection studies included 3 groups of animals; 4 control animals received 200 mg polyclonal human IgG, 6 animals received 20 mg CD4-IgG2 and 7 animals received 200 mg CD4-IgG2 (Fig. 1). All 4 control animals were infected with a peak viremia at 1–3 weeks of approximately 10^7^–10^8^ viral copies/ml. Of the 20 mg dose CD4-IgG2 treated animals, 3/6 animals were protected. Two of the infected animals showed a course of infection very similar to control animals whereas a third showed a delayed primary viremia in that virus was only detectable at week 3. Two of the protected animals showed a “blip” of virus at week 2 but no virus was detectable at later time-points (up to 23 months). Of the 200 mg CD4-IgG2-treated animals 5/7 were protected. The two infected animals showed a primary infection course similar to control animals and the 5 protected animals showed no indication of infection at any time-point subsequent to challenge.

### Pharmacokinetics of CD4-IgG2 in protection experiments

**Figure 2:**
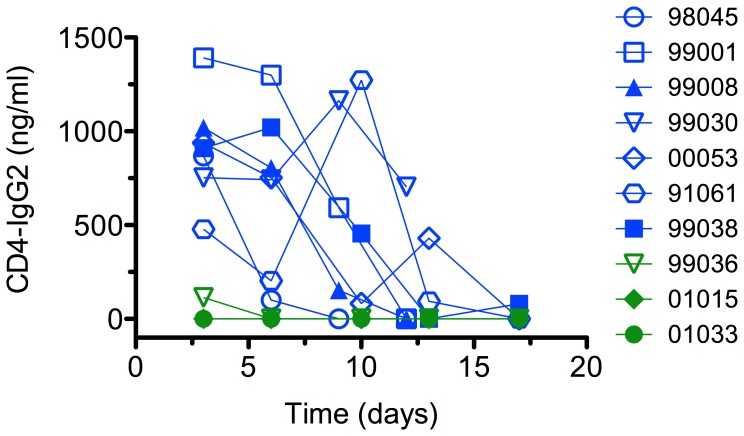
Plasma concentration of CD4-IgG2 in treated animals. Animals administered 200 mg of CD4-IgG2 showed plasma concentrations in the range of 500 to 1400 ng/ml at the time of challenge. No apparent correlation between plasma concentration and protection was observed. The CD4-IgG2 concentration at the time of challenge in animals administered 20 mg was 100 ng/ml for 1 animal and below the limit of detection (8 ng/ml) for 2 animals. Serum samples from the remaining 3 animals administered 20 mg were unavailable for this analysis.

The plasma concentrations of CD4-IgG2 in macaques receiving the 20 mg dose were below the level of detection (8 ng/ml) at the time of challenge (day 3) for 2 animals and 100 ng/ml for one animal (Fig. 2). Unfortunately, the plasmas of three of the animals were not available for study (see Material and Methods). Serum neutralization was undetectable at a 1∶4 dilution for the 3 sera available in a U87 cell pseudovirus assay. For animals given the 200 mg dose, plasma concentrations ranged between 500 and 1400 ng/ml at the time of challenge and in most, but not all, animals decayed weakly in the first 3 days after challenge and then more precipitously (Fig. 2). Again no serum showed any measurable neutralization at a 1∶4 dilution in a pseudovirus assay.

### Development of macaque anti-CD4 responses

**Figure 3:**
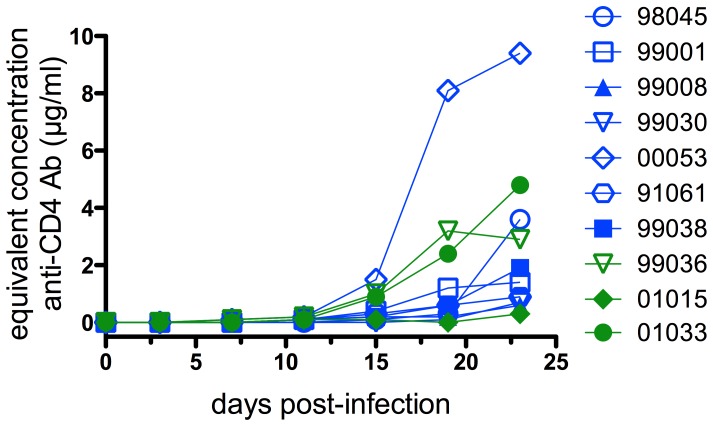
Anti-human CD4 response in animals treated with CD4-IgG2. Animal sera were tested in a human CD4-specific ELISA to detect macaque antibody responses against CD4-IgG2. Serum samples were tested up to 23 days post-viral challenge and no responses were detected before day 15, indicating that the animal protection outcome was independent of a response against human CD4. Serum samples from 3 animals administered 20 mg CD4-IgG2 were unavailable for this analysis.

The sera of animals were examined for the development of macaque anti-human CD4 responses out to 23 days post challenge. Anti-CD4 responses were not detected before 10 to 15 days post challenge and only in some animals (Fig. 3). The area under the curve (AUC) of the anti-human CD4 response for protected (1.015 days×anti-human CD4 response, log) and non-protected animals (0.7215 days×anti-human CD4 response, log) were not significantly different (two-tailed Mann Whitney U, p = 0.34), suggesting no association between the magnitude of this response and the outcome of viral challenge.

## Discussion

Perhaps the most favored animal model of HIV is SIV infection of macaques. In particular, the ability of immunogens to protect against high-dose SIVmac239 (or the highly related SIVmac251) challenge of macaques has been viewed as a stringent test of the potential utility of such immunogens as vaccines in humans [27]. There have been many failures of vaccines based on eliciting cellular immunity (“T cell vaccines”) to protect against high-dose SIVmac239 challenge [28] and some limited successes [e.g.29], [30]. Here we show that a high degree of protection can be achieved against high-dose SIVmac239 challenge at low serum concentrations and neutralizing titers of the antibody-like molecule CD4-IgG2. This molecule is not strictly an antibody but nevertheless it likely operates in vivo largely by preventing viral entry in a similar fashion to antibody as discussed below and therefore the result is an encouraging one for the possible benefits of humoral immunity in protection against HIV exposure.

How may CD4-IgG2 protect so effectively? In the first instance, it is unlikely that the level of protection results from macaque anti-human CD4 antibodies. First, significant titers of such antibodies at very variable levels only began to develop at day 10–15 when infection was well established in the control and treated non-protected animals. The kinetics of SIV infection strongly suggest that the critical phase to prevent or abort infection is likely to be in the first few days following challenge prior to the “broadcast” phase at 7–10 days when the virus disseminates through the lymphatic system [31]. Second, there was no association between levels of serum anti-CD4 antibodies and protection (Fig. 3).

CD4-IgG2 neutralizes SIVmac239 very efficiently in vitro. This is not necessarily in conflict with the oft-used label for SIVmac239 of “ highly neutralization resistant”. In fact that label is given because natural SIVmac239 infection elicits rather modest serum neutralizing titers, which may reflect a low immunogenicity of neutralizing epitopes on the SIVmac239 envelope rather than an intrinsic resistance to neutralization. Indeed, the sensitivity of SIVmac239 to CD4-IgG2 neutralization argues this to be the case. Despite the sensitivity of SIVmac239 to CD4-IgG2 neutralization in vitro, the serum concentrations of CD4-IgG2 achieved in vivo in the protection experiments were relatively low leading to low predicted neutralizing titers (undetectable in 1∶4 dilution). Nevertheless, the serum concentration of CD4-IgG2 did seem to correlate with protection, as animals treated with 200 mg (5 out of 7 protected) had a measurable level of CD4-IgG2 in the serum compared to animals treated with 20 mg (3 out of 6 protected, 2 of the protected showed a viral blip), which suggests an association between serum CD4-IgG2 concentration and protection. Interestingly, protection in the presence of low antibody neutralizing titers has been reported for macaques passively administered the broadly neutralizing monoclonal antibody (bnMAb) 2G12 and challenged with SHIV [2], [7]. In contrast, protection against SHIV challenge by a polyclonal antibody or the bnMAb b12 was associated with relatively high serum antibody neutralizing titers [3], [5], [10]. It is worth noting, that the above-mentioned studies were performed with a single administration of a monoclonal IgG1 antibody or a polyclonal antibody preparation. A direct comparison to the present study is therefore not without caveats as we used an antibody-like molecule and a continuous dosing apparatus. Nevertheless, the main conclusion being drawn is that a neutralizing agent, antibody or antibody-like molecule, can induce protection against SHIV or SIV challenge in vivo and in the case of CD4-IgG2 at a very low serum concentration.

Protection against SIVsmE660 has been associated with low levels of serum neutralizing antibodies in macaques [14] following immunization, although this was low-dose repeated rather than high-dose SIV challenge. Vaccine protection against low-dose repeated SIVmac251 challenge has also been associated with serum anti-Env antibodies, although without detectable neutralization [15]. This circumstance mirrors somewhat that reported for protection in the RV144 human vaccine trial [32], [33].

A number of factors beyond classical neutralization have been identified that may contribute to effective antibody protection against SIV/HIV challenge. One factor is the ability of antibody to interact with Fc receptors [8], [9], which presumably facilitates infected cell killing and/or phagocytosis of infected cells or virions and/or virus immobilization by Fc receptor-bearing effector cells. However, CD4-IgG2 bearing the γ2 heavy chain binds poorly to human activating Fcγ receptors and is not expected to be efficient at these effector functions [18]. A second factor is the ability of antibody to bind to neonatal Fc receptors (FcRn) on cervical reserve epithelial cells that may provide protection at mucosal surfaces as recently proposed for live attenuated protection against SIV challenge (Li, Haase et al, manuscript submitted). The γ2 Fc of CD4-IgG2 is expected to bind well to FcRn. Finally, we cannot rule out that unique properties of CD4-IgG2, perhaps related for example to the induction of gp120 shedding and irreversible neutralization are responsible for the unusually effective protective activities of this molecule against SIV challenge.

As mentioned, the use of live attenuated SIVmac239 delta Nef immunization has been one of few successful approaches to provide protection against heterologous SIV challenge in rhesus macaques [16]. Our study was not a vaccination study in a classical sense. However, it is, to our knowledge, the first direct demonstration that protection can be achieved against SIVmac239 by a neutralizing antibody or antibody-like molecule. Previously clinical studies have shown that CD4-IgG2 is well tolerated in humans but utility could be limited by a very short half-life [24], [25], [26]. An alternative delivery strategy to bypass a continuous dosing is the approach taken by Johnson and colleagues, who immunized macaques with AAV vectors carrying an immunoadhesin (rhesus CD4 fragment attached to the Fc fragment of rhesus IgG2) [34]. In their study, a long lasting serum neutralization titer and complete protection against subsequent SIVmac316 challenge were achieved. Although the technology is still in its infancy and not without its limitations, the approach may be as a viable alternative to traditional immunization strategies and CD4-IgG2 could be a candidate molecule alongside traditional IgG antibodies.

In conclusion, our studies show protection against high-dose challenge by the prototype SIVmac239 virus at very low levels of a neutralizing agent in challenge experiments and therefore should stimulate further studies on the conditions for protection in this important animal model of human HIV infection.

## Materials and Methods

### Macaques

This study was carried out in strict accordance with the recommendations in the “Guide for the Care and Use of Laboratory Animals” of the National Institutes of Health. All protocols for male Indian rhesus macaques were reviewed and approved by the Institutional Animal Care and Use Committees of the Scripps Research Institute and the Graduate School of the University of Wisconsin (Animal Welfare Assurance No. A3368-01). The animals were housed in accordance with the Association for Assessment and Accreditation of Laboratory Animal Care Standards. To ensure the health of the nonhuman primates assigned to this study, each animal was evaluated twice daily by an animal research technician or veterinary technician for the evidence of disease or injury. Animals were fed chow twice daily and produce enrichment (fruits, veggies, seed, and nuts) once daily. Environmental enhancement that involves foraging opportunities was provided 5 times per week. As part of preparation for the experiments, animals were removed from their usual housing facilities and moved into facilities dedicated to SIV research. Animals were not single housed for prolonged periods of time (>2 weeks) before SIV infection. Once infected, animals were singly housed to prevent cross contamination of SIV infection and spread of opportunistic infections. Viral challenge and sample collections were performed under ketamine or ketamine/medetomidine induced anesthesia, and all efforts were made to minimize pain and distress. At the start of all experiments, all animals were experimentally naïve and were negative for antibodies against HIV-1, simian immunodeficiency virus (SIV), and type D retrovirus. The decision to euthanize an animal depended on several issues, in addition to progression to AIDS, and included weight loss of 20% of total body weight, infection with opportunistic pathogens and no response to treatment after 7–10 days, infection with opportunistic or pathogenic organisms and progressive decline in condition regardless of treatment and time course, chronic diarrhea and inappetence, neurological signs such as disorientation, abnormal gait or posture, tremor etc. Any deteriorating condition deemed to be particularly distressful to the animal as assessed by the veterinary staff is a condition for euthanasia. Animal were euthanized by an IV overdose (50 mg/kg) of sodium pentobarbital, preceded by up to 15 mg/kg of ketamine IM.

### CD4-IgG2 administration and viral challenge

CD4-IgG2 was administered by a subcutaneously implanted ALZET® osmotic pump to maintain its delivery over a two-week period [35]. More than 50 references have been published using this drug delivery method without reporting any adverse effect. Three days after the start of immunoglobulin administration animals were challenged with 3–5×10^3^ TCID_50_ SIVmac239 intrarectally.

### Challenge virus

SIVmac239, GenBank accession no. M33262 was a generous gift of Dr Ron Desrosiers (New England Primate Research Center). Plasma viral load quantifications were performed by branched-DNA assay. The threshold of sensitivity was >125 vRNA copies/ml (Bayer Diagnostics, Berkeley, CA).

### Immunoadhesin

CD4-IgG2 was prepared in recombinant Chinese hamster ovary cells and purified to >95% homogeneity by column chromatography as described previously [18]. Purified material was stored at −80°C prior to use.

### Neutralization assays

Neutralization of CD4-IgG2 was assessed by 3 different methods. (i) PBMC primary isolate neutralization assay. Neutralization of the primary isolate SIVmac239 was performed using phytohemagglutinin (PHA)-activated peripheral blood mononuclear cells (PBMCs) from a single rhesus macaque (no. 355) as target cells. Cells from this animal replicate SIV efficiently. Neutralization assessment was carried out as described previously [5]. (ii) Pseudovirus assay using U87 target cells. Viruses pseudotyped with SIVmac239 envelope and carrying the luciferase reporter gene were generated by cotransfection of 293T cells with the pNL4-3.luc.R-E- and SIVmac239-env vectors. Pseudovirus neutralization was assessed by measuring infection of CD4^+^CCR5^+^ U87 target cells (obtained from the ARRRP, contributed by Hong Kuy Deng and Dan Littman) using the luciferase reporter gene [36]. (iii) LTR SEAP neutralization assay using CEMx174(T1)-SIV-SEAP target cells. CEMx174(T1)-SIV-SEAP cells contain a SEAP reporter gene under the control of a Tat-inducible SIVmac239 LTR. Pseudovirus SIVmac239 infection and neutralization were measured by SEAP activity in cell-free culture supernatant as described previously [37]. Serum neutralization titers were determined using the pseudovirus/U87 assay. Unfortunately, samples from 3 of the 20 mg treated animals were misplaced during a major laboratory relocation and were unavailable. Available samples were from both infected and protected animals.

### Serum anti-CD4 measurements

Ninety six-well ELISA plates (Corning, Acton, Ma) were coated overnight at 4°C with 50 µl of recombinant soluble CD4 at 2 µg/ml in PBS (pH 7.5). Plates were washed twice with ELISA wash buffer and then saturated with 100 µl of 3% BSA in PBS for 1 h at room temperature (RT). After ten washes, serial dilutions of serum from CD4-IgG2-treated and control animals or of standard anti-CD4 antibody were added, and incubated for 2 h at RT. The plates were washed ten times and bound antibody was detected using a goat anti-human IgG conjugated to alkaline phosphatase (Pierce, Rockford, Il) in PBS for 1 h at RT. After extensive washing, detection was carried out with p-nitrophenyl phosphate tablets (Sigma-Aldrich, St Louis, MO) following the supplier's recommendations.

### Statistical analyses

Areas under the curve (AUCs) and Mann-Whitney tests for anti-human CD4 responses were calculated using GraphPad Prism for Mac, version 5.0a (GraphPad, San Diego, CA). AUCs were log-transformed before the analysis.
